# Discussion on Same

**Published:** 1893-12

**Authors:** 


DISCUSSION.

The discussion of the paper was opened by Dr. Mary Weeks Burnett, of Chicago; Dr. C. J. Lewis, of Chicago; Dr. Henry Hulst, of Michigan; Dr. Duncan, of Chicago, and the Chair following. The discussion was as follows:

Dr. Mary Weeks Burnett:

I am exceedingly interested in the subject of the paper. The sex question, with all that pertains to it of good and of evil, has not yet had the search-light of science thrown upon it. Physicians and jurists, more than any others, have brought to them diseased and depraved conditions for treatment, and it is a duty they owe to humanity at large that the results of their researches for remedies should be made known, and the best methods for elevating and refining the race be freely discussed. I know of but one society as yet established for the study of all problems associated with the sex question—The National Woman Physicians' Association, organized in 1888. We are making special investigation into questions of marriage, motherhood, and the relation of the sexes. I am sure that our Association will be interested in this paper, and much in sympathy with the method spoken of for the reduction of crime.

Dr. C. J. Lewis:

The subject matter of the paper is one of great importance. I would suggest, however, that the principle upon which the good results are looked for would be not strictly on the line of heredity, so-called, but rather that new members coming by birth into the state should not be allowed such criminal environments. Taking away the environment will remove the cause. That is, have no members—children—grow up in such environment. I think the principle of heredity has been somewhat overestimated at the expense of acquirement of conduct from association. I

am in hearty accord with the general sentiment of the paper, and earnestly hope it will stimulate others to join in the effort of establishing among our people a sentiment, the application of which will enable all persons to effectually control the impulsive expression of the sexual instinct.

Whether the sexual instinct is of the brain, or whether its center is in the lumbar region of the spinal chord, is, perhaps, a question. It is customary to speak of the sexual sense as having its center in the spinal chord, and from which center, this center—the chord—emanate all reflex actions. If we hold that the center for this sense is in the spinal chord, then the mind can have but little control over it—which is probably true. If a physician should find the offending seminal organs were beyond the control of the mind in any given person, this fact could be taken as a basis for advising their removal. But before he could do this, it would require the development of a public sentiment that would crystalize in such legislation as would permit the physician to proceed, when complying with the privileges bestowed, to carry out to the fullest extent whatever he should conclude to be for the best interests of both patient and State alike, whatever operation the exigencies of the case might require. From this point—view—what the doctor contends for in his paper is timely, and fitting for all who have an uncontrollable sexual impulse.

Dr. Henry Hulst:

I was very much surprised that Von Schenck Notzing's book was not mentioned in this paper. It is upon this subject of sexual perversion, but the author advocates something very different from castration as a method of dealing with it. He employs psycho-therapeutics, and his results demand attention. His constitutes, in my opinion, the correct method of dealing with these unfortunates. The following is the title of the book, now well-known to every student of neurology: "*Die Suggestions Therapie bie Krankhaften Erscheinungen des Geschlechtsinnes*," etc., by Dr. A. Freiherrn Von Schenck Notzing, Stuttgart, 1892. My friend, Prof. Chaddock, who translated the Psychopathia Sexualis, by Von Krafft-Ebing, has also, I believe, made a translation into English of the former work.

Moll, of Berlin, published a book on it, in 1891—a work known everywhere. I will content myself by merely referring to these works.

Dr. Duncan:

I was much interested in this paper, for various reasons. Coming from a physician, we would naturally suppose he would suggest a remedy, but I think the remedy suggested would not prove as effectual as has been supposed. I am inclined to believe that we will have to come, by and by, to taking care of this class of humanity, just as lepers are taken care of, and not allow them to run at large. They have not mental nor moral control of themselves; therefore, the State ought to take care of them.

So far as the relation of sexual perversion is concerned, especially in children, it is not usually due to the sexual sense, masturbation, but to irritation, and here is a fact that should have a very important bearing on the legal aspect of cases of this kind. I'm very glad the subject has been brought up, for I think it ought to be discussed; that we should reach out and enlist public sentiment in favor of it. It is easy to enact a law, but unless the people support it by popular endorsement, it becomes a dead letter.

The President, Mr. Clark Bell, called Dr. H. M. Bannister to the chair, and said:

"This is a subject which has lately attracted thoughtful minds, in many directions. I will endeavor to confine myself to the legal aspects of the questions involved. We have a little shrinking (due to training) from a free discussion of this question. That is a misfortune. It is a question we should not shrink from discussing. In the South, where Dr. Daniel lives, it is a burning question. All of us remember the case of the progeny of a woman in the State of New York that was traced out, who had given birth, in her life-time, to over fifty descendants that were criminals. That which was traced in New York could have been traced elsewhere in a similar way. It is a grave problem, where the State, which is responsible for the welfare of its people, under the law, has not the power to protect itself from progeny certain to be criminal. We kill the rattlesnake, to try to compass its extinction and prevent its propagation. I think most superintendents of asylums would be willing to recommend that it would be unwise in all respects to allow insane persons to intermarry, if the question of progeny was to be considered. I am inclined to differ with Dr. Daniel upon the legal questions he raises in this matter, and the legal authorities he cites. There is a difference between a surgeon's bleeding a patient, and the operation of castration. The crime of mayhem could be charged against the one, and not upon the other. In the absence of legislative action, I think very few physicians would be willing to take the responsibility of castration, unless there should be such a disease of the organs as threatened the patient's life, or serious injury, when, of course, castration would be perfectly legitimate and proper; but, to castrate merely for the purpose named by the physician to prevent procreation, would be beyond the physician's province, and would be (in all of the States of the American Uaion, in which I am familiar with the law), a violation of the law, and would constitute the crime of mayhem. I am rather in sympathy with Prof. Krafft-Ebing's views on this subject, and with those of the author cited by Dr. Hulst. There can be no doubt whatever of the legal right of the State to pass laws to make this act a punishment for crime, and to insert this penalty in the methods of punishment in the category of punishment for crimes. On that there can not be the slightest difference among lawyers and jurists. The question narrows itself, in my judgment, to the single one of the propriety of its exercise.

I think the question might be submitted in this form: Would the race be benefited by the extirpation of the power of procreation, in the male, in cases where there was reason to believe that danger to the race would follow reproduction, or in cases of those criminals who had violated the law, as suggested by the author of the paper, thus placing themselves within the power of the State to inflict any punishment it saw fit? Under the old law rape was punishable by death, and certainly this is lesser in its effects than taking life. To contrast it with our knowledge of its operation and effect upon animals, we know it would be beneficial, I think, placing the question as suggested by the author of the paper, in this way, would leave publicists, and jurists, and political economists, in favor of a wise law for the restricted exercise of this power, in a punitory sense, for crime by the sexual pervert, as well as a judicious use of it for the insane."

[From transactions of the Congress, in Medico-Legal Journal for September; the paper itself appears in the Medico-Legal Journal for December.—Ed.]

